# Metronidazole Based Floating Bioadhesive Drug Delivery System for Potential Eradication of *H. pylori*: Preparation and In Vitro Characterization

**DOI:** 10.3390/polym14030519

**Published:** 2022-01-27

**Authors:** Faiza Naseem, Shefaat Ullah Shah, Sheikh Abdur Rashid, Arshad Farid, Mazen Almehmadi, Saad Alghamdi

**Affiliations:** 1 Skin/Regenerative Medicine and Drug Delivery Research, GCPS, Faculty of Pharmacy, Gomal University, Dera Ismail Khan 29050, Pakistan; faizanaseem17@gmail.com (F.N.); shefaatbu@gmail.com (S.U.S.); 2 Gomal Center of Biochemistry and Biotechnology, Gomal University, Dera Ismail Khan 29050, Pakistan; arshadfarid@gu.edu.pk; 3 Clinical Laboratory Sciences Department, College of Applied Medical Sciences, Taif University, Taif 26571, Saudi Arabia; 4 Laboratory Medicine Department, Faculty of Applied Medical Sciences, Umm Al-Qura University, Makkah al-Mukarramah 24382, Saudi Arabia; ssalghamdi@uqu.edu.sa

**Keywords:** metronidazole, direct compression, swelling index, total floating time, agitational intensity

## Abstract

Metronidazole has the potential to produce local stomach specific action in order to treat *Helicobacter pylori* induced peptic ulcer disease. The current project executes the development of osmotically controlled bioadhesive metronidazole loaded effervescent floating tablets with optimized floating and swelling behavior. Direct compression technique was used to prepare the tablets. The designed formulations exhibited physico-chemical properties within acceptable optimum limits as per pharmacopeial requirements. The results of tablet floating studies revealed that all formulations, except F1 and F5, had good buoyancy characteristics (TFT > 12 h except F2 and F8 with TFT of 6 h). Formulation F2 containing guar gum in higher concentration with carbopol and formulation F8 containing guar gum in 50% decreased concentration in combination with HPMC and carbopol had enhanced FLT appreciably, with least TFT as compared to formulations F3, F4, and F6 (ANOVA; *p* ≤ 0.05). Formulation batches of F3, F4, and F6 exhibited appreciable FLT as well as TFT and were optimized formulations. Out of the above mentioned optimized batches, F4 and F6 formulations showed low FLT (4 and 5 s respectively). The results of the swelling study indicated a proportionate increase in the swelling index with increase in time. A significantly higher swelling ratio was found with formulation F6 and F4 compared with that of F7 and F8 (ANOVA; *p* ≤ 0.05). Additionally, the impact of pH change, agitational intensity, as well as increasing concentration of NaCl was investigated on drug release. It was observed that agitational intensity had no effect on drug release rate while increasing concentration of NaCl produced an increased drug release from the dosage form as compared to the drug release exhibited by the formulations in the absence of NaCl. Overall, this project could have valuable contribution in the fabrication of metronidazole loaded effervescent floating tablets. Gastro-retentive systems are expected to enhance local stomach specific action of anti *H. pylori* agents based on their buoyancy and swelling behavior.

## 1. Introduction

Drug administration through the oral route is considered as the most appropriate, feasible, and the most convenient mode as far as the delivery of drugs is concerned, with numerous benefits such as safety, self-administration, cost economical, non-invasive, and improved patient compliance [1]. However, the oral route is sometimes associated with different issues that are major obstacles, e.g., patterns of gastric emptying, minimum drug residence, failure of drug targeting in specific area, and negligible drug release [2].

Metronidazole has bactericidal effect and is efficacious to kill microorganisms, either facultative anaerobes or obligate anaerobes. It sits right in the cusp among medications selected for the management of Helicobacter pylori, i.e., it constitutes an important member of triple regimen therapy as an active adjunct [3,4]. Metronidazole is a class 1 type of drug in the biopharmaceutical classification system. It undergoes absorption in the stomach and possesses pH-independent effects [5,6]. Conventionally, metronidazole exhibits a rapid and almost complete absorption when given orally [7].

Helicobacter is a Gram-negative, facultative anaerobe which produces gastric mucosal irritation leading to chronic inflammation. Different people may show different episodes of inflammatory lesions which vary in intensity appreciably [8,9]. This microbe constitutes about 50% of the world population epidemiologically, with over 90% of infected individuals being residents of developing countries [10,11,12].

Floating drug delivery systems have paramount applications to achieve stomach specific action or local targeting of drug in the stomach. Such systems are divided into two sub-categories, i.e., effervescent and non-effervescent systems [13,14]. Effervescent floating systems are made up of matrix of polymeric material of hydrophobic, expandable or polysaccharide nature with additional sodium bicarbonate or other gas-generating systems. Carbon dioxide generation due to reaction with the gastric acidic contents is a major determinant of floating behavior of such systems. Non-effervescent systems, on the other hand, are chiefly composed of hydrophilic colloids that exhibit swelling upon reaction with the gastric contents, resulting in lowering of the specific density that allows upward movement of the fluid producing variations in the absence of gas production [14,15]. The suggested criteria for drugs to be formulated as floating drug delivery system include local action of drugs in stomach [16], drugs showing absorption window in stomach or upper part of the intestine [17], and candidates exhibiting poor solubility or instable nature in intestinal fluid [18]. Floating drug delivery systems are peculiar to induce prolonged drug buoyancy in the stomach without having any specific effect in gastric emptying time alteration. This results into desired retarded drug release rate from the system with prolonged gastric retention time, thus provides good control of drug plasma concentration variations [19]. However, one major drawback or limitation of such systems is the provision of sufficient fluid saturation of stomach in order to keep the dosage form buoyant. For this issue, bioadhesive polymers are incorporated, e.g., carbopol, HPMC, dextran, tragacanth, chitosan, sodium alginate, polyethylene glycol, polyacrylic acid, and polylactic acid [13]. These polymers stick to gastric mucosal lining due to their natural, peculiar behavior. Their implication in dosage form development is responsible to cause improved bioavailability of drugs having an absorption window in the upper part of the GIT; as a result, frequency of drug administration is also reduced [8]. Mucoadhesive delivery systems are designed to solve the issue of GIT transit time reduction. Such polymers are effective enough to enhance the dosage form adhesiveness owing to their bioadhesive potential. These polymers have beautified the literature consisting of both natural as well as synthetic mucoadhesive polymers [20].

The current study was undertaken to develop and optimize the metronidazole based osmotically controlled bioadhesive floating drug delivery system by incorporating various muco-adhesive polymers based on buoyancy and bio-adhesion concepts. The aided benefits would be mirrored to offer *H. pylori* induced peptic ulcer healing along with improved patient compliance owing to a decreased administration frequency.

## 2. Materials and Methods

### 2.1. Materials

Metronidazole was kindly gifted by Ferozsons Laboratories, Nowshehra, KPK, Pakistan; HPMC K4M, carbopol, guar gum (Dow Chemical Company, Midland, MI, USA), chitosan (high molecular weight) and sodium alginate were used as bio-adhesive polymers, microcrystalline cellulose employed as diluent, talc as glidant, magnesium stearate (Sigma-Aldrich, Co., St. Louis, MO, USA) as lubricant. Sodium bicarbonate, purchased from a local market, was incorporated as gas generating agent. HCl and instruments needed for project execution were obtained from Research Laboratory on demand. All the chemicals employed were purified and of analytical grades.

### 2.2. Pre-Formulation Studies

Pre-formulation studies represent an initial step to develop dosage form rationally. There is an obvious focus on physical and chemical properties of novel compound that could have a strong impact as far as drug performance and efficacious development of dosage form is concerned [21]. Pre-formulation activity is done solely to formulate dosage form via rational approaches, to attain maximum probability of success in order to achieve acceptable product, and finally to provide sound background to reach towards product optimization [22].

#### 2.2.1. Construction of Standard Calibration Curve

Stock solution (1 mg/mL) of metronidazole was prepared by dissolving 100 mg of metronidazole in 100 mL of 0.1 N HCl. From stock solution, various dilutions ranging from 20 µg/mL up to 50 µg/mL were prepared. These sets of dilutions were subjected to analysis on UV spectrophotometer (UV-1800 SHIMADZU, Kyoto Japan) to record their absorbance values at λ_max_ 277 nm for metronidazole. To construct standard calibration curve, absorbance values were plotted against respective concentrations. Triplicate readings were recorded as mean ± SD.

#### 2.2.2. Metronidazole Solubility Analysis

Saturated solubility experiment was carried out to determine solubility of metronidazole in various solvents at different pH media. Saturated solutions of metronidazole were casted by adding an excess quantity of drug (1 gm) to 10 mL portion of selected solvents sealed in closed containers. They were subjected for 24 h at 25 ± 1 °C and 37 ± 1 °C on shaking water bath. Aliquots from clear supernatant portion were withdrawn after specified time (24 h) and by using distilled water sufficiently diluted to analyze spectrophotometrically at 277 nm λ_max_ [21].

#### 2.2.3. Micromeritical Properties

Among pre-compression characteristics of metronidazole decorated floating tablets, angle of repose, compressibility index, and Hausner’s ratio were calculated. The above-mentioned flow properties of various formulations of metronidazole based floating matrices play significant role to determine the product’s appropriateness to be used as gastro-retentive system.

- Angle of Repose

Angle of repose denotes frictional forces between the loose powder bed or granules [23]. Metronidazole loaded formulations powder material in sufficient quantities were passed through a funnel on the horizontal surface from a 2 cm height until heap is formed that touch the funnel tip. Both radius as well as height of powder heap were measured. The angle of repose was calculated by the following equation:

(1) tan θ=h/r

(2) $$\theta =\tan^{-1}h/r$$

where:

θ = angle of repose
h = pile height
r = radius of the circle [24]

b. Compressibility Index and Hausner’s Ratio

The flow behavior of prepared powder can be determined by compressibility index and Hausner’s ratio that are simple and popular methods, although very close to each other [25]. These methods required bulk as well as tapped density and compared with the rate at which it is packed down. To note bulk density, the formulation powder was poured into a graduated cylinder followed by surface leveling with a spatula to note the bulk volume. To obtain the tapped volume, the graduated cylinder was tapped 100 times [26]. Equation (3) gave us the value of the powder bulk density:

(3) $$\mathrm{Density}\left(\rho \right)=m/v$$

where:

m = powder mass either bulk or tapped
v = bulk or tapped volume

Equations (4) and (5) are used for the determination of Hausner’s ratio and compressibility index, respectively [27].

(4) Hausner’s Ratio=ρ tapped/ρ bulk

(5) $$\mathrm{Compressibility}\;\mathrm{Index}\left(\%\right)=\left(\rho \;\mathrm{tapped}-\rho \;\mathrm{bulk}/\rho \;\mathrm{tapped}\right)\times 100$$

where:

ρ tapped = tapped powder density
ρ bulk = bulk powder density

#### 2.2.4. Drug Excipients Compatibility Studies

Excipients careful selection has key role to obtain stable, effective and successful solid dosage form formulation. The drug excipients compatibility is primed in order to obtain effective, safe, and stable product. The main goals of compatibility studies involve exploring suitable excipients having compatibility with active drug and no stability issues of active ingredients, assigning each excipient risk level relative to their functional class [28].

ATR-FTIR spectra were obtained using an ATR-FTIR spectrometer (Spectrum 100, Perkin Elmer, Shelton, CT, USA) using MIRacle ATR accessory (PIKE Technologies, Madison, WI, USA). The samples included pure metronidazole, polymers, and physical mixtures of drug polymer formulations obtained after crushing floating tablets of each batch. Recording of scans was performed from 4000 cm^−1^–625 cm^−1^. The scan period was 12 min and the resulting spectra were thus compared for any spectral changes [3].

### 2.3. Formulation Development and Optimization

Osmotically controlled metronidazole based controlled release floating tablets were formulated. The quantity of drug was kept constant, but varying amounts of polymers either alone or in a blend were used in different formulation batches. Preparation of pilot batches was done consisting of 100 tablets of each formulation type. Metronidazole as well as other ingredients of formulation were weighed individually by using a digital electronic balance (AX 120, SHIMADZU, Kyoto, Japan). Accurately weighed portion of osmotically controlled polymer, hydrophilic polymer, hydrophilic gums, gel forming polymers, and muco-adhesive polymers along with microcrystalline cellulose were placed in a mortar and subjected to geometric mixing. To this mixture, a pre-weighed portion of metronidazole was added and thoroughly mixed. The resultant mixture was sieved through a mesh size 40, followed by their collection in plastic bag and an additional 5 min of blending. The requisite portion of NaHCO_3_ was incorporated, followed by an additional 5 min mixing. At last, magnesium stearate and talc in sufficient quantity were added. The final blend of mixture was again passed through a sieve number 40 followed by thorough mixing for 10 min, and compressed into tablets by employing a single punch tablet machine (Erweka-Apparatebau compression machine type T B-24). Tablet hardness was kept constant at 6.5 ± 0.33 kg/cm^2^ [7].

To optimize osmotically controlled bioadhesive floating matrices of metronidazole, the following parameters were kept constant in all eight formulation batches:

- Tablet hardness more than 5.5 kg/cm^2^
- Tablet friability less than 0.8%
- Floating lag time not more than 120 s
- Buoyancy duration more than 6 h

### 2.4. Post-Compression Evaluation Parameters

#### 2.4.1. Physical Appearance and Dimensional Specifications

Physical appearance as well as shape of compressed tablets were examined under magnification. The dimensional specifications of prepared batches of floating tablets included thickness and diameter. Vernier caliper (Erweka GmbH, Langen, Germany) after thorough calibration was used to measure thickness and diameter of tablets. At least five tablets were randomly selected from each formulation and individually subjected to thickness and diameter measurement [3]. Then, the average or mean values of the dimensional specification test were expressed in the form of mean ± SD.

#### 2.4.2. Hardness and Friability of Floating Tablets

Hardness tester (Erweka Model TB 24 Apparatus, Langen, Germany) was used for hardness calculation. Randomly selected five floating tablets from each batch were subjected to hardness testing. The hardness value was expressed in kg/cm^2^ unit [29]. Roche friabilator was used to determine friability of prepared tablets. Randomly, ten tablets were selected from each formulation batch and weighed to obtain W1. These tablets were then placed in the friabilator and the apparatus was run for 4 min at 25 rpm. After 4 min treatment or 100 revolutions, the tablets were again weighed to obtain W2. The following Equation (6) was used to calculate the friability expressed in percentage:

(6) $$\%\mathrm{Friability}=\left(W1-W2/W1\right)\times 100$$

The acceptable pass limit of friability was considered to be less than 1% [30].

#### 2.4.3. Uniformity of Weight Test for Floating Tablets

From each formulation batch, randomly, twenty tablets were taken and individually weighed on weighing balance (AX 120, SHIMADZU, Kyoto, Japan). For each batch of formulations, an average weight was calculated as mean ± SD. The allowable percentage variation in tablet weights is given in Table 1 below [3].

#### 2.4.4. Drug Content Determination

Randomly, ten tablets were chosen for drug content determination. They were made into powder form and powder mass equivalent to 643 mg of tablet weight was weighed and dissolved in 100 mL 0.1 N HCl. The sample was dissolved by continuous stirring at 37 ± 0.5 °C temperature for 5 min. It was then subjected to filtration process by passing through a 0.45 mm Whatman filter paper to obtain a filtrate. Then, metronidazole content was determined spectrophotometrically at 277 nm by using UV spectrophotometer (UV-1800, SHIMADZU, Kyoto, Japan) by correlating with the previously generated standard calibration curve. Similarly, standard solution of metronidazole was prepared, and percent drug content was noted by using the following Equation (7) [31].

(7) $$\%\mathrm{Drug}\;\mathrm{Content}=\left({\mathrm{Sample}}_{abs}/{\mathrm{Standard}}_{abs}\right)\times 100$$

At least triplicate readings were taken and averaged.

#### 2.4.5. Impact of pH

The pH change method was employed to investigate the effect of pH and to obtain the performance reliability of developed optimized formulations independent of the pH parameter. In this method, the release behavior of optimized formulations was noted as per the protocol of pH change method. This pH change method was utilized to note drug release in order to mimic the GIT transit time and pH. The pH change method involved for first two h, simulated gastric fluid (pH 1.2) as release media followed by next two h, acetate buffer (pH 4.5), and lastly, by replacing with simulated intestinal fluid (pH 6.8) up to the remaining time frame of 24 h. Since it is impossible to rule out the release mechanism as well as kinetics of drug release via the pH change method and transit time, therefore, it is preferred to find the drug release at pH 1.2 for the maximum period of 24 h [32]. The requisite dissolution media, i.e., 0.1 N HCl (pH 1.2), acetate buffer (pH 4.5), and simulated intestinal fluid (pH 6.8) were prepared (900 mL each) as per USP specifications, and kept at 37 ± 1 °C. the dissolution apparatus was run at 100 rpm. At pre-decided time intervals, an aliquot of 5 mL was taken, followed by its passage through a 0.45 µ membrane filter and analyzed at 277 nm by a UV spectrophotometer (UV-1800, SHIMADZU, Kyoto, Japan) [21].

#### 2.4.6. Impact of Agitational Speed

To note the effect of agitational intensity of 0.1 N HCl as well as other release media such as acetate buffer (pH 4.5) and simulated intestinal fluid (pH 6.8), the dissolution apparatus was set at different rotational speeds to determine the release behavior of the optimized formulation. USP method 1 (rotating basket) was employed in the dissolution apparatus at 50 and 100 revolutions per minute. Similarly, at a pre-determined time, samples were taken and analyzed at 277 nm after filtering through a 0.4 µ membrane filter [21].

#### 2.4.7. Impact of Osmotic Pressure

Media having different osmotic pressures were used to conduct release behavior experiments of optimized formulations in order to obtain confirmation about drug release mechanism. An osmotically active solute (NaCl) was incorporated in simulated gastric fluid (0.1 N HCl maintained at 37 ± 1 °C) in order to increase osmotic pressure of the media. This experiment was switched on at 100 rpm with 0.5% and 0.9% sodium chloride concentration. At a predetermined time interval, 5 mL samples were withdrawn, followed by replacement with an equal volume of fresh solvent maintained at same temperature to keep the volume of the vessel constant. In order to determine the drug concentration of withdrawn samples, absorbance was taken by a UV spectrophotometer (UV-1800, SHIMADZU, Kyoto, Japan) at 277 nm wavelength [33].

#### 2.4.8. Floating Behavior Evaluation

- Swelling Index

Weight gain or water uptake experiment was used for evaluation of swelling index determination of formulated floating tablets. Tablets selection from each batch of formulation was made randomly, weighed individually (Wo), and were placed in separate beakers containing approximately 50 mL of 0.1 N HCl kept at 37 ± 0.5 °C. The tablets were removed after 2 h regular time intervals up to a maximum of 8 h time period. The tablets were priorly air-dried, followed by re-weighing to obtain Wt. The following equation was used for water uptake or percent swelling index or weight gain.

(8) $$\%\mathrm{Swelling}\;\mathrm{Index}=\left(\mathrm{Wt}-\mathrm{Wo}/\mathrm{Wo}\right)\times 100$$

where:

Wt = tablet weight at “t” time
Wo = initial weight before immersion [34]

b. In vitro Buoyancy/Floatability Study

This important parameter of floating drug delivery system was determined by employing a USP type 2 dissolution apparatus (paddle method). The tablets were placed into glass jars filled with 0.1 N HCl up to 900 mL volume with rotation speed set at 50 rpm. The apparatus was constantly maintained at 37 ± 0.5 °C temperature. The experiment ran for 24 h. The time taken by the tablets to come to the surface of dissolution medium as well as the time duration for which tablets constantly afloat on dissolution media were noted. These were known as floating lag time and total floating time, respectively. Triplicates were carried out to obtain an average result shown as mean ± SD [28].

c. Tablet Density

For floating tablets, density represents as crucial parameter, because tablets will only afloat when their density is less than the density of gastric contents (1.004 g/cm^3^) [35]. By using the following Equation (9), floating tablets density was determined:

(9) ρ=m/v

where:

ρ = density; m = mass of the tablet in gram
v = tablet volume in cm^3^

Equation (10) depicts calculation of volume:

(10) $$v={\pi r}^{2}h$$

where:

r = radius of tablet in cm
h = tablet crown thickness in cm

#### 2.4.9. In Vitro Drug Release/Dissolution Test Studies

USP type 2 dissolution apparatus was used to carry out in vitro drug release experiment by using 900 mL of 0.1 N HCl solution maintained at 37 ± 0.5 °C temperature. The rotations were set at two different levels, i.e., 50 rpm and 100 rpm and representative samples from all the batches were subjected to expose towards dissolution medium. At the pre-selected time period, an aliquot of about 5 mL was withdrawn from the vessel and simultaneously replenished with same volume of dissolution medium (0.1 N HCl) maintained at constant temperature of 37 ± 0.5 °C. The samples were filtered through 0.5 mm Whatman filter paper followed by their analysis on UV spectrophotometer (UV-1800 SHIMADZU, Kyoto 604-8511, Japan) at 277 nm. The percent drug release was calculated with the help of standard calibration curve already generated during pre-formulation studies. Triplicate readings were taken to obtain an average and are shown as mean ± SD [36].

#### 2.4.10. Dissolution Profile Kinetics

In vitro dissolution test represents itself an important and useful tool in process of drug development over recent years. It is regarded as crucial parameter in quality control and under particular situations can act as surrogate to describe the bio-equivalence assessment to predict bioequivalence. For modified release dosage forms, guidelines suggest the USP dissolution apparatus type 1, 2, 3, or 4 as this equipment gives satisfactory outcomes. However, novel advancements in dissolution apparatus currently available or having new agitation profile, media variation and keeping the samples in the media without altering release mechanism, require careful planning. To establish accuracy in dissolution test, it is worthy to note that the release mechanism knowledge as well as physico-chemical properties of active ingredient are of utmost importance. Release mechanisms could be described by a variety of linear as well as non-linear kinetic models. Similarly, test and reference dissolution profiles could also be compared by using such kinetic models, i.e., zero order kinetics, first order kinetics, Higuchi model, and Korsmeyer–Peppas equation.

The drug release data obtained from osmotically controlled metronidazole based floating tablets was fitted in the power law by using the Microsoft Excel Software [37]. The following equation, called power law equation, was used to determine the drug release mechanism:

Power Law

(11) $$\mathrm{Mt}/M\infty ={\mathrm{Kt}}^{n}$$

where:

Mt/M∞ = drug release fraction after “t” time
K = rate constant; n = release exponent

when

n = 0.5, it means drug release occurs by Quasi–Fickian diffusion mechanism
n > 0.5, then release mechanism exhibited by drug will be non-Fickian, anomalous or case Ⅱ
n = 1, it depicts case Ⅱ or zero order kinetics [38]

### 2.5. Statistical Analysis

All the experiments were performed in triplicates and averages expressed as mean ± SD. One way ANOVA was applied for the statistical analysis. A *p*-value < 0.05 was considered significant.

## 3. Results

### 3.1. Standard Calibration Curve

**Figure 1:**
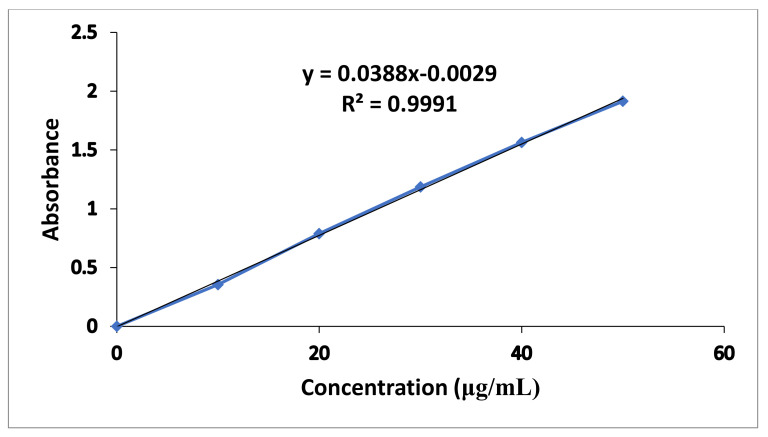
Metronidazole Standard Calibration Curve in 0.1 N HCl.

The standard calibration curve of metronidazole in 0.1 N HCl presented in Figure 1 exhibited good linearity with R^2^ value of 0.9991. The regression equation of the graph was y = 0.0388x − 0.0029.

Where “y” represents slope while “x” reflects unknown concentration of drug.

### 3.2. Solubility Studies

Table 2 reflects metronidazole solubility studies in various solvents such as water, 0.1 N HCl, phosphate buffer (pH 4.5, 6.8 and 7.4) kept at 25 °C and 37 °C. It is obvious from the table that metronidazole offered elevated solubility in water as compared to other remaining solvents where it exhibited lower solubility profile.

### 3.3. Micromeritical Characteristics

Table 3 show flow characteristics for effervescent metronidazole based floating tablets. Calculated values of angle of repose fall in the range of 25.3°–31.6° for all the formulation batches prepared. This range of values exhibited excellent flowability of the mixture, except F7 having good flowability. This might be due to the presence of very fine and low molecular weight microcrystalline cellulose incorporated in excess quantity in formulation F7, because this batch of formulation contained only osmotically controlled polymer carbopol 934P and hence, a larger amount of MCC and lactose was added to obtain the desired weight of formulation. The results of compressibility index of all the formulations (F1–F8) indicated that the resultant mixtures could easily be compressed when floating tablets were made by direct compression method. The compressibility index values ranged from 9.18–13.91%. The compressibility index value increases when there is decreased concentration of guar gum in the formulation batches (F2, F5, F8) as there was 50% reduction in the guar gum concentration in formulations F5 and F8 as compared to formulation F2. Formulations F5 and F8 were adjusted with other polymers to compensate 50% reduction in concentration of guar gum. It is the same case with compressibility index values for formulation batches F1 and F5; F3 and F6; F4, F6 and F8 containing chitosan, sodium alginate, and HPMC, respectively, either alone or in admixtures with other polymers. The compressibility index value of F4 ≤ 10 indicated better compressibility as compared to other formulation batches. The results of Hausner’s ratio showed good flow properties exhibited by various formulation batches. The range obtained for Hausner’s ratio was 1.10–1.15. It is worth mentioning that alginate containing formulations alone (F3) or admixtures with HPMC (F6) produced better flow properties as compared to the rest of the formulation batches. Prior to compression, magnesium stearate was incorporated, acting as lubricant, to minimize the risk of powder sticking to the punches of compression machine [39].

### 3.4. Drug Excipients Compatibility Studies

**Figure 2:**
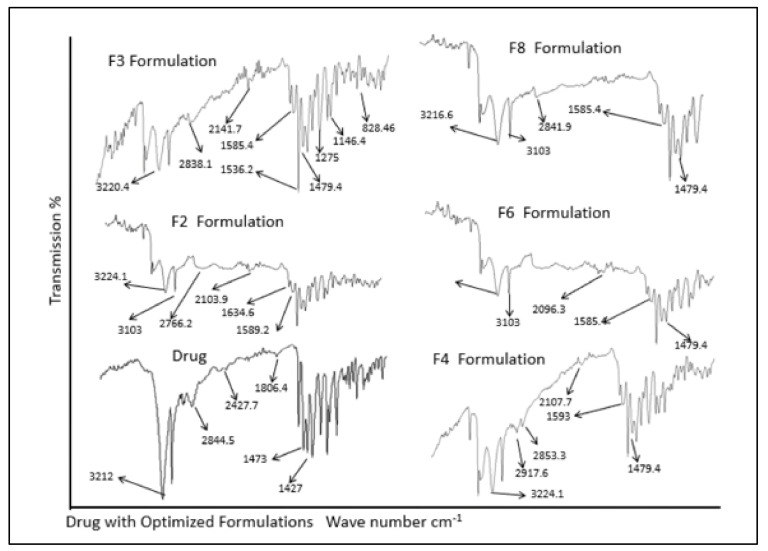
ATR-FTIR spectra of drug and formulations.

For drug excipients compatibility studies, ATR-FTIR analysis was done for pure drug, various polymers and physical mixtures of drug and excipients. The analysis of ATR-FTIR spectra confirmed the absence of significant interactions between model drug and polymers as shown in Figure 2. Absorption bands at 3212 cm^−1^ indicate O–H stretching, 2844 cm^−1^ (C–H stretching), 1806 cm^−1^ (N–O stretching), 1473 cm^−1^ (NO_2_ symmetric stretching), and 1427 cm^−1^ (C–H bending in plane) were assigned as fingerprints of metronidazole. It was observed that the prominent band peaks of the drug and polymers were not significantly altered as described previously [3]. Similarly, ATR-FTIR spectra of drug in physical mixture with polymers exhibited all the prominent characteristic peaks as found in the spectra of individual metronidazole, sodium alginate, chitosan, guar gum, HPMC, carbopol 934 P, and various other excipients excluding the possibility of any possible interaction. The data found was in accordance with previous findings [40].

### 3.5. Preparation of Osmotically Controlled Floating Tablets of Metronidazole

Direct compression method was employed to develop osmotically controlled metronidazole based floating bioadhesive tablets by incorporating various polymers used in different ratios as described in Table 4. However, polymers used in the formulations such as guar gum, chitosan (high molecular weight), sodium alginate, and HPMC were incorporated either alone (150 mg) representing 23% of total formulation weight and 1:0.75 drug to polymer ratio or their physical admixture with other polymers in the concentration of 75 mg each representing 12% of total formulation weight and 1:0.375 drug to polymer ratio. Formulation batch F7 comprised only of osmotically controlled polymer carbopol 934 P did not contain any other polymer, rather contained 100 mg lactose and 125 mg MCC, and was used as reference formulation to compare the effects of other formulations.

### 3.6. Metronidazole Floating Tablets Evaluation

#### 3.6.1. Physical Appearance and Dimensional Specifications

Under microscope, tablets appeared circular shaped having no cracks. The dimensional specifications (thickness and diameter) of floating tablets were found to be within the acceptable range as shown in the Table 5. The mean thickness of formulated floating tablets was almost similar in all eight formulations and fell in the 3.42 mm–3.70 mm range. Similarly, the tablet diameter was found to be in the range of 15.10 mm–15.28 mm when measured by using Vernier caliper.

#### 3.6.2. Hardness of Floating Tablets

The hardness values of all the formulations from this study showed this parameter to be within the specified limit of 5–10 kg/cm^2^, as shown in Table 5. An increased hardness value may produce variations in floating lag time whereas, decrease in hardness has an obvious effect on dissolution profile [41].

#### 3.6.3. Friability of Floating Tablets

The friability of metronidazole loaded floating tablets of the formulations (F1–F8) was presented in Table 5. No cracked, broken, or split tablets were found at the end of the friability test. The results of friability values of all the formulations were within the official compendial limits (<1%). Thus, this ensured the mechanical stability of floating tablets.

#### 3.6.4. Weight Variation Study of Floating Tablets

All formulations (F1–F8) complied with the weight uniformity test accurately, as there was not more than ± 5% deviation of any individual tablet weight from its respective mean value. The results of tablet uniformity of weight of all the formulation batches complied with official compendial specifications are presented in Table 5 [42].

#### 3.6.5. Drug Content Determination/Content Uniformity Test

The results obtained for content uniformity of all the formulation batches (F1–F8) were fitted in the official compendial criteria and found within the acceptable range, because the individual contents of all formulation batches fell within the acceptable official range of 85–115%. The obtained results were in the range of 95.21–99.03% for metronidazole. Our results are in agreement with previous reported findings [7,19].

#### 3.6.6. Floating Behavior Evaluation

The results of tablet floating studies as shown in Table 6 revealed that all formulations, except F1 and F5, had good buoyancy characteristics (total floating time more than 12 h except F2 and F8 with total floating time of 6 h) as their density is lower than that of GI fluids. Formulations F1 and F5 exhibited no floating properties due to gel formation. The gel may be produced due to the presence of high molecular weight chitosan along with carbopol in F1 and combination of chitosan, carbopol and guar gum in F5 due to their sticking properties [43]. Formulation F2 containing guar gum in higher concentration with carbopol and formulation F8 containing guar gum in 50% decreased concentration in combination with HPMC and carbopol had enhanced floating lag time appreciably with least total floating time as compared to formulations F3, F4, and F6 (ANOVA; *p* less than 0.05). The responsible factors might be HPMC K4M viscosity as well as decreased guar gum concentration which resulted in significantly prolonged lag time [44]. Formulation batches of F3, F4, and F6 exhibited appreciable floating lag time as well as total floating time and were optimized formulations. Out of the above mentioned optimized batches, F4 and F6 formulations showed low floating lag time (4 and 5 s respectively). The findings of our study are in close agreement with the previous reported studies [3,45].

#### 3.6.7. Swelling Index Determination

**Figure 3:**
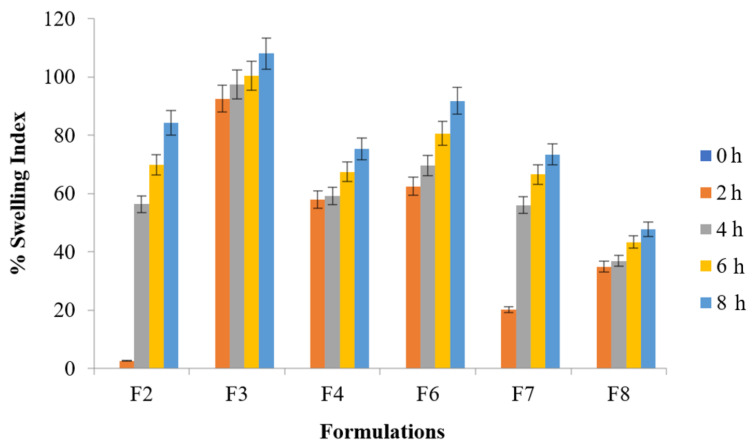
% Swelling index of metronidazole tablet (*p* < 0.001) F3 vs. F8 (*p* < 0.05) F3 vs. F2, F4, and F7.

**Figure 4:**
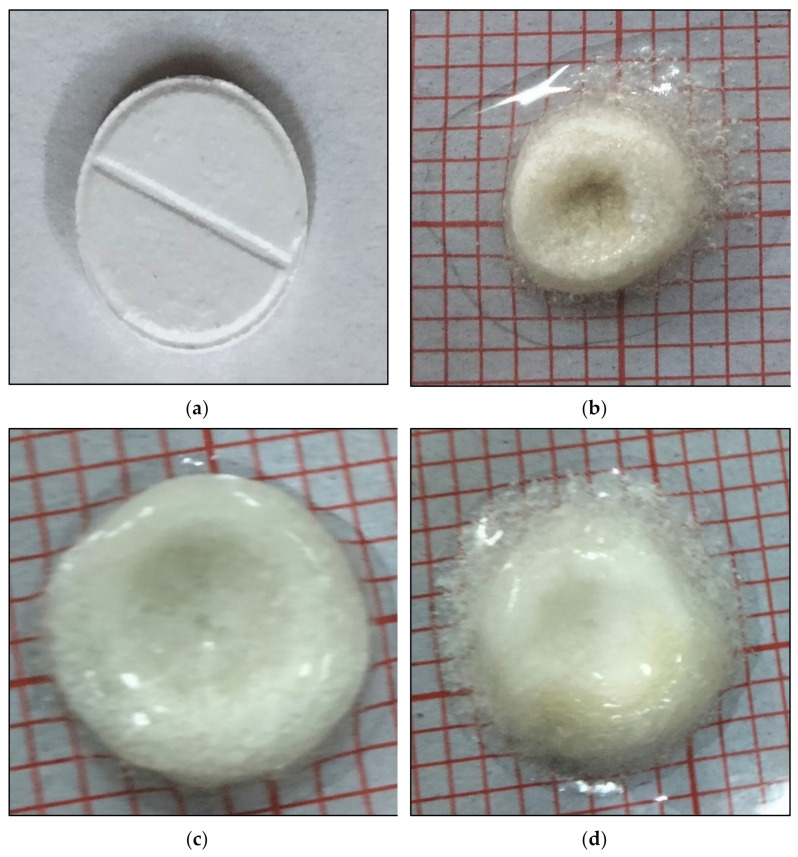
Swelling behavior of effervescent floating tablet of metronidazole (F3) (**a**) tablet at 0 h, (**b**) tablet swelling after 2 h, (**c**) tablet swelling after 4 h (**d**) tablet swelling after 8 h.

The selection from all the optimized formulation batches was made to perform this test up to 8 h indicating tablet swelling up to 108% of their original size (F3 formulation). Significantly higher swelling ratio was found with formulation F3 than with that of F8 (ANOVA; *p* ≤ 0.001) whereas ANOVA; *p* ≤ 0.05 was found for F3 vs. F2, F4, and F7. Significantly higher swelling ratio was found with formulation F6 and F4 than with that of F7 and F8 (ANOVA; *p* ≤ 0.05). Figure 3 explained water uptake behavior of the floating tablets whereas Figure 4 illustrated the water uptake behavior of optimized formulation F3 at various time intervals.

All the formulations were incorporated with NaHCO_3_ as gas generating agent. The tablets afloat and remained buoyant for hours. The total lag time as well as total floating duration for all the optimized formulations are presented in Table 6. It was noted that F4 and F6 containing HPMC alone and in combination with sodium alginate produced prominent swelling and had good water uptake capabilities than other optimized formulations (F2, F7 and F8) containing guar gum, carbopol, lactose, etc. [46]. The same trend exhibited by F4 and F6 prepared with HPMC alone or in combination with sodium alginate had better compressibility followed by retarded floating behavior. These results are in agreement with the findings of other authors who manipulated flow behavior and compaction of different grades of HPMC [29].

#### 3.6.8. Tablet Density

All prepared optimized formulations floated on the surface of any fluid that mimic gastric contents (0.1 N HCl, pH 1.2, temperature 37 ± 1 °C), thus ensuring that density kept in required value. The data for tablet density are given in Table 6.

#### 3.6.9. In Vitro Drug Release Behavior

To study drug release from metronidazole loaded osmotically controlled floating tablets, the following dissolution conditions were set as presented in Table 7.

**Figure 5:**
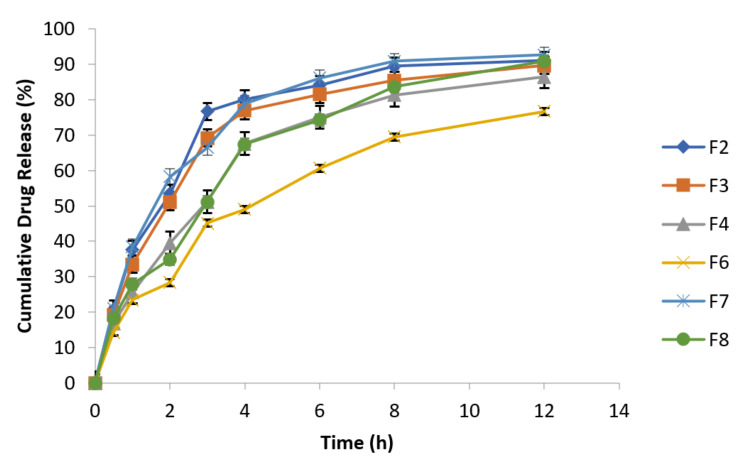
Release behavior of effervescent floating tablets of metronidazole at 100 rpm (n = 3).

At specified time intervals, as mentioned in the above Table 7, samples were taken followed by replacement with equal volume of fresh dissolution medium kept at the same temperature and analyzed spectrophotometrically at 277 nm. The data of cumulative % drug release are presented in the following Figure 5.

F2 and F8 exhibited 91.09% and 90.85% drug release after 12 h, respectively, while F7 (reference formulation) showed 92.71% drug release. F2 formulation in 1:1 concentration of guar gum and carbopol has resulted reduction in burst drug release with improved sustained release behavior [47]. Formulation F6 has best retardant potential as shown by the drug release data (76.69%) because it is composed of combination of polymers (carbopol, sodium alginate and HPMC). Similarly, F3 (89.69%) and F4 (86.44%) formulations also exhibited sustained release behavior and retarding potential of these formulations is due to the presence of sodium alginate and HPMC respectively along with osmotically controlled polymer carbopol 934 P in 1:1 concentration. The optimized formulations are F4 (HPMC K4M alone) and F6 (HPMC K4M in combination with sodium alginate). Formulation F6 exhibited 76.69% drug release which has more retarding potential as compared to formulation F4 that exhibited 86.44% drug release.

#### 3.6.10. Effect of Agitational Intensity on Drug Release Data

**Figure 6:**
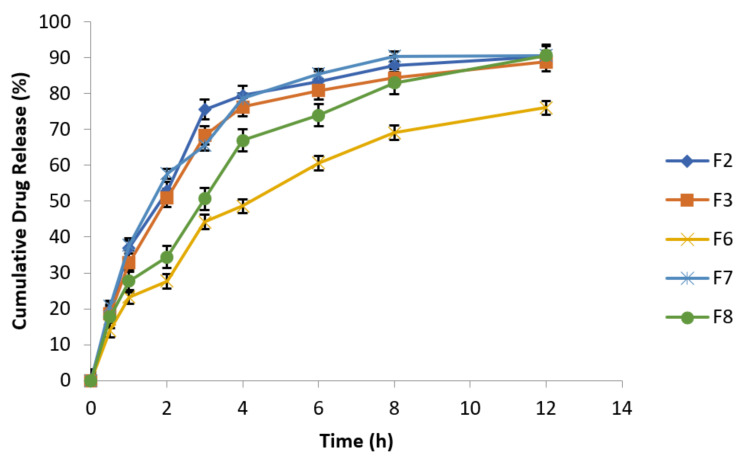
Release behavior of effervescent floating tablets of metronidazole at 50 rpm (n = 3).

To investigate the impact of agitational intensity upon drug release, the apparatus was set at 50 rpm rotational speed and all the other parameters were kept constant, as mentioned in Table 7. The release studies of all the optimized formulations were performed in USP Type Ⅰ dissolution apparatus at different rotational speeds (50 and 100 rpm). Figure 6 clearly reflected that release profile from optimized formulations of metronidazole based floating tablets was absolutely not dependent on agitational intensity of release media. Hence, it can be assumed and expected that the optimized fomulations will remain independent of hydrodynamic conditions developed naturally at the absorption site [48].

#### 3.6.11. Effect of pH on In Vitro Drug Release

**Figure 7:**
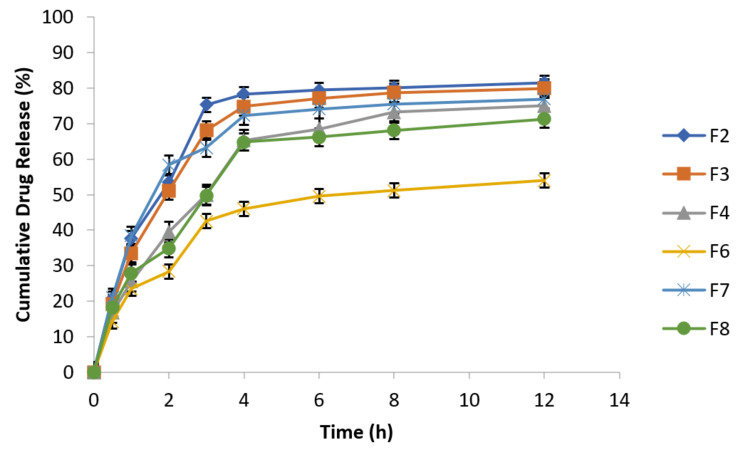
Release behavior of metronidazole effervescent floating tablets at different pH values (n = 3).

To investigate pH effect on drug release from metronidazole loaded osmotically controlled floating tablets, the USP type 1 method was used at 100 rpm by using 0.1 N HCl (pH 1.2 for first 2 h) followed by acetate buffer (pH 4.5 for next 2 h) and finally by employing simulated intestinal fluid (pH 6.8) for remaining 8 h. Temperature of the experiment was kept constant at 37 ± 1 °C. At specified time intervals (0.5, 1, 2, 3, 4, 6, 8, and 12 h) samples were taken followed by replacement with equal volume of fresh dissolution medium kept at same temperature and analyzed spectrophotometrically at 277 nm. The data of cumulative % drug release are presented in the following Figure 7.

#### 3.6.12. Effect of Osmotic Pressure on Drug Release

**Figure 8:**
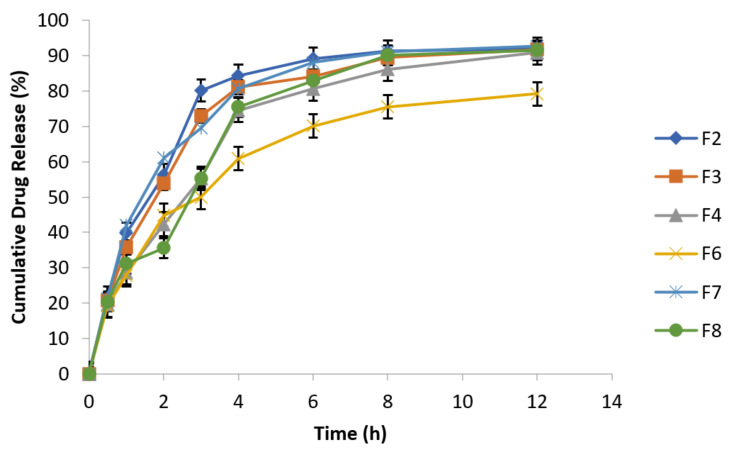
Effect of osmotic pressure (0.5% NaCl) on drug release at 100 rpm in 0.1 N HCl (n = 3).

**Figure 9:**
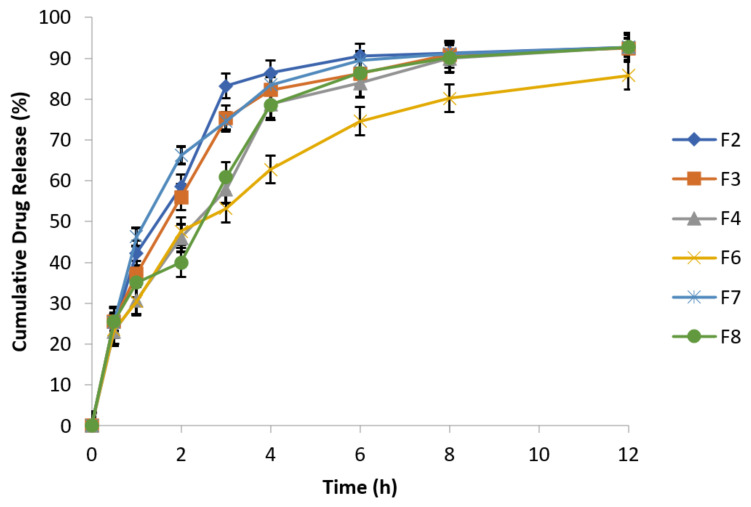
Effect of osmotic pressure (0.9% NaCl) on drug release at 100 rpm in 0.1 N HCl (n = 3).

Results showed that metronidazole drug release increased in release media containing NaCl concentrations of 0.5% and 0.9%, which is evident from Figure 8 and Figure 9. Metronidazole is a water soluble drug; therefore, matrix swelling rate was rate determining step in its release.

#### 3.6.13. In Vitro Drug Release Kinetic Profiling

In this work, the Korsemeyer–Peppas model was employed to investigate mechanism of drug release from the developed floating system. This model is utilized to present polymeric release when the release mechanism is not well known or when more than two different release mechanisms could be involved.

Our drug release data, when fitted into the Korsemeyer–Peppas equation, exhibited anomalous non-Fickian drug release mechanism. Formulations F4, F6, F7, and F8 exhibited the best fitted release model showing anomalous non-Fickian diffusion mechanism (Table 8 and Table 9). The data of NaCl incorporation in 0.5% and 0.9% concentration were fitted into Korsemeyer–Peppas model, then the formulations described the Fickian diffusion mechanism (Table 10 and Table 11). This might be due to decrease in swelling as evident from previous literatures that NaCl addition resulted into decreased swelling as well as increased drug release by diffusion mechanism [33].

## 4. Discussion

The current study utilized a metronidazole effervescent floating system with the additional advantage of swelling properties to keep an increased gastric stay. Various formulations were designed and evaluated containing novel blends of polymeric admixtures in order to obtain the objectives of the current study. The standard calibration curves were employed to determine drug contents in cumulative drug release. The ionization constant (pKa) of metronidazole was 2.62 indicating the basic nature of the drug; that is why a lower solubility profile was exhibited by metronidazole at higher pH values. Moreover, it is also found that by increasing the temperature, the solubility of the drug also increased as a general rule; hence, at elevated temperature (37 °C), metronidazole solubility was also enhanced in all the solvents. This may be due to a heat absorption phenomenon, because the majority of drugs possess heat of solution in positive integer values that can result in an improved solubility profile whenever there is elevation in temperature of the system. All the estimated parameters of micromeritical properties such as angle of repose, compressibility index, bulk and tapped densities as well as Hausner’s ratio were found within the acceptable limits and found suitable for direct compression method to formulate the metronidazole loaded floating drug delivery system. The bulk and tapped densities were calculated in order to determine Hausner’s ratio as well as compressibility index. The values for both types of densities for various formulation batches were almost similar with slight variations only that were statistically insignificant. The relative close proximity of calculated values of both types of densities indicates less significant inter-particulate interactions and powder mass of various formulation batches is free flowing. The values of compressibility index and Hausner’s ratio exhibited good flow properties for the powder mass [49]. ATR-FTIR analysis was carried out to investigate the possibility of any type of interaction among active ingredient and various excipients. Although the results revealed compatibility between active drug and various excipients excluding the risk of incompatibility, however, formulation processing might also result in changes into functional groups as well as chemical changes; therefore, this aspect of floating tablets was also excluded [50]. Many trials were made to develop floating tablets and finally, eight formulations were designed. Each formulation batch out of a total of eight formulations contained a constant quantity of metronidazole (200 mg) as well as osmotically controlled polymer carbopol 934 P (150 mg), representing a drug to carbopol ratio of 1:0.75 (23%). Direct compression technique was selected owing to manufacturing ease, less time consuming aspect, as well as affordable low cost. Thickness and diameter of floating tablets play a prominent key role, because either decreasing or increasing any one of them may have a direct impact on tablet density. An increased tablet thickness may result in the alteration of tablet floating behavior, whereas decreased tablet thickness may have an impact on floating lag time, thus the mean value of both these parameters should be less than the density of gastric contents (1.004 g/cm^3^). Although official pharmacopeial specifications do not exist for tablet hardness, tablets should neither be too hard nor too soft. The acceptable limits for tablet crushing strength, as suggested by some literatures, ranged from 5–10 kg/cm^2^ for uncoated tablets. Floating tablet of metronidazole is not meant to disintegrate since it exhibited controlled release behavior and if hardness has no impact on other parameters, particularly dissolution and release profile, then it could be well accepted. However, tablet hardness measurement is not a reliable indicator as some formulations undergo capping or lamination on attrition even when they are compressed very hard; as a result, the tablets undergo fragmentation, chipping or made into powder form [51]. As a general rule, when hardness of the tablet increased, the friability or percent mass loss in the formulation decreased proportionally therefore, by keeping greater compression force can modify the results of friability test to follow the USP limits [52]. The content uniformity test helped to ensure achieving of dosage form uniformity, because the active ingredient metronidazole constitutes a large tablet part, unlike in cases where formulations contained potent drugs available in low doses and greater part of the tablet is formed by the excipients. The floating behavior of the tablets may significantly change owing to increase or decrease in the concentration of incorporated polymers. Both low density as well as low gelling capability of polymers help the tablet to stay afloat by gas entrapment in the gel network. The presence of NaHCO_3_ as a gas generating system (effervescent agent) decreased the floating lag time in the optimized formulations. Swelling ratio or water uptake showed the quantity of water uptake by the polymers. The swelling appeared to be due to ionization of functional groups as well as functional network structure. There was proportionate increase in swelling index with increase in time. The reason behind this outcome might be the incorporation of hydrophilic polymers (HPMC, sodium alginate, guar gum, carbopol 934 P). Initially, polymer swelling occurs in the outermost layer which could produce gel layer as a barrier. As the outermost gel layer barrier slowly dissolved, then there was swelling of new gel layer due to water uptake. This process is repeated continuously towards newly exposed surfaces. This phenomenon is responsible for dosage form integrity maintenance and facilitated the controlled drug release profile. The viscosity of polymers directly affected swelling index, matrix integrity, and floating behavior. It was found that swelling process and polymer viscosity existed in linear relationship. The presence of NaHCO_3_ produced effervescence when tablets were exposed to the media that mimic gastric contents (0.1 N HCl, pH 1.2) maintained at 37 ± 1 °C. To offer floating behavior, it is important to note that tablet density should be less than that of gastric contents (1.004 g/cm^3^). Formulation F7 acting as reference formulation is composed of only osmotically controlled polymer carbopol 934 P without any addition of other hydrophilic polymers such as guar gum, HPMC, and sodium alginate, was designed to note the difference in drug release rate as compared to other formulations which additionally contained hydrophilic polymers either in equal concentration to that of carbopol or 50% reduced concentration. It was observed that release of metronidazole from all floating tablets was significantly dependent upon the concentration of retardant polymers incorporated in the formulation. There was exhibition of decreased drug release from floating tablets when retardant material was present in increased amount. It was observed that HPMC K4M, due to less viscosity and low molecular weight, could not maintain matrix integrity sufficiently when used alone and did require sodium alginate combination to control the drug release. It is important to study matrix integrity of the floating tablets, because lack of tablet physical integrity could result into smaller fragments after being broken down and an escape from GIT [29]. Drug release from floating tablets up to larger extent does not depend on agitational intensity or rotational speed of the release media. Sometimes, floating tablets move to the intestine as observed in some cases [53]. Therefore, a study should be conducted by taking into account physiological pH of varying sections of GIT. Thus, the study of drug release from optimized formulations was carried out by keeping in view the pH as well as transit time attributes of GIT. This was executed at pH 1.2 for two hours (0.1 N HCl), pH 4.5 for next two hours (acetate buffer), and pH 6.8 for the remaining time period (simulated intestinal fluid). It could be observed that controlled release behavior was exhibited by metronidazole at pH 1.2 and 4.5. This was mainly due to the presence of osmotically controlled polymer carbopol 934 P and other hydrophilic polymers present either alone or in combination. At pH 6.8, due to substantially reduced swelling of the floating tablets, the observed drug release was unpredictable. This might be due to the fact that metronidazole, being weakly basic drug, remained unionized at higher pH value, leading to obvious reduced solubility, thus resulting into unpredictable drug release [54]. To note the effect of osmotic pressure on release studies, optimized formulations were exposed to media having different osmotic pressures. Drug release patterns were investigated for metronidazole incorporated into optimized formulation batches in release media containing different NaCl concentrations (0.5% and 0.9%). By increasing the concentration of NaCl, there was proportionate increase in drug release rates whereas, proportionate decrease in swelling rates. It was observed that diffusion path length and resistance were responsible to determine the release profile of metronidazole from optimized formulations when exposed to media containing different amounts of NaCl. However, swelling index as well as barrier gelled layer decreased proportionally when the NaCl concentration in the media was increased. This resulted into increased diffusion rate. The gel barrier layer in 0.1 N HCl was thicker than that in media containing NaCl. This exhibited slower drug release rate in 0.1 N HCl and increased drug release in NaCl containing media [33]. The quantification of drug release data becomes easy to interpret when in vitro dissolution profiles are fitted in the mathematical models. The release data were fitted into the Korsemeyer–Peppas model in which exponent (n) values specify drug release mechanism. For a cylindrical tablet, if n ≤ 0.45, it specifies Fickian diffusion or Case Ⅰ transport. If n value is more than 0.45 but less than 0.89, then it specifies non-Fickian diffusion or anomalous release, i.e., release by diffusion and polymer relaxation. An exponent value of 0.89 indicates zero order kinetics or Case Ⅱ transport (release by erosion).

## 5. Conclusions

This study discourses the formulation and evaluation of osmotically controlled floating tablets of metronidazole. The addition of polymers such as carbopol, HPMC K4M, guar gum, sodium alginate, and gas generating agent sodium bicarbonate was responsible to achieve in vitro buoyancy. Formulation F6 showed a preferred drug release profile up to 12 h following anomalous non-Fickian diffusion. An increased drug release was found by the incorporation of NaCl in release media as compared to media lacking NaCl. Thus, the conclusion of this research work clearly points out to a promising potential of this metronidazole floating prolong release dosage form as a substitute to the conventional dosage form for the management of *H. pylori* induced peptic ulcer disease.

## Figures and Tables

**Table 1:** Allowed % age deviation limits in tablet weight variation.

Tablets Weight	Allowed Variation (%)
Average weight 130 mg or less	±10
More than 130 mg up to 324 mg	±7.5
Above 324 mg	±5

**Table 2:** Metronidazole solubility studies conducted at different pH media (mean ± SD).

Prescribed Solvents	Solubility (mg/mL) at 25 °C	Solubility (mg/mL) at 37 °C
Water	9.235 ± 1.135	10.568 ± 1.637
0.1 N HCl	3.315 ± 1.983	3.973 ± 1.996
Phosphate Buffer pH 4.5	0.0231 ± 0.0012	0.0276 ± 1.0023
Phosphate Buffer pH 6.8	0.0356 ± 0.0017	0.0365 ± 0.0019
Phosphate Buffer pH 7.4	0.0388 ± 0.0014	0.0425 ± 0.0021

**Table 3:** Flow properties of metronidazole loaded effervescent floating tablets.

Batch Code	Angle of Repose	Bulk Density (gm/mL)	Tapped Density (gm/mL)	Compressibility Index (%)	Hausner’s Ratio
F1	27.6 ± 0.04	0.438 ± 0.05	0.497 ± 0.01	11.45 ± 0.06	1.12 ± 0.04
F2	28.11 ± 0.01	0.433 ± 0.11	0.4871 ± 0.05	10.71 ± 0.03	1.12 ± 0.02
F3	25.6 ± 0.02	0.435 ± 0.06	0.4861 ± 0.02	10.57 ± 0.05	1.13 ± 0.02
F4	25.3 ± 0.05	0.438 ± 0.01	0.4971 ± 0.08	9.18 ± 0.03	1.13 ± 0.07
F5	28.1 ± 0.08	0.439 ± 0.05	0.5091 ± 0.04	12.13 ± 0.16	1.15 ± 0.05
F6	25.8 ± 0.02	0.432 ± 0.10	0.4751 ± 0.02	11.52 ± 0.03	1.10 ± 0.02
F7	31.6 ± 0.08	0.443 ± 0.03	0.5160 ± 0.05	13.48 ± 0.01	1.15 ± 0.07
F8	26.5 ± 0.03	0.438 ± 0.04	0.5041 ± 0.05	13.91 ± 0.03	1.12 ± 0.04

Data shown as mean ± SD (n = 3).

**Table 4:** Composition of floating tablets of metronidazole.

Ingredients	F1 (mg)	F2 (mg)	F3 (mg)	F4 (mg)	F5 (mg)	F6 (mg)	F7 (mg)	F8 (mg)
Metronidazole	200	200	200	200	200	200	200	200
Carbopol 934P	150	150	150	150	150	150	150	150
Chitosan	150	-	-	-	75	-	-	-
Guar Gum	-	150	-	-	75	-	-	75
Sodium alginate	-	-	150	-	-	75	-	-
HPMC	-	-	-	150	-	75	-	75
MCC	75	75	75	75	75	75	125	75
Lactose	-	-	-	-	-	-	100	-
NaHCO_3_	60	60	60	60	60	60	60	60
Talc	5	5	5	5	5	5	5	5
Magnesium stearate	2.5	2.5	2.5	2.5	2.5	2.5	2.5	2.5

**Table 5:** Physico-chemical parameters of metronidazole effervescent tablets.

Batch Codes	Hardness (kg/cm^2^)	Friability	Thickness (mm)	Diameter (mm)	Weigh Variation (mg)	Drug Content (%)
F1	5.4 ± 0.25	0.41 ± 0.02	3.56 ± 0.015	15.11 ± 0.006	648 ± 4.12	95.21
F2	5.8 ± 0.29	0.78 ± 0.21	3.68 ± 0.032	15.11 ± 0.063	647 ± 4.23	98.09
F3	6.2 ± 0.10	0.83 ± 0.11	3.42 ± 0.010	15.13 ± 0.023	651 ± 3.87	98.63
F4	6.3 ± 0.45	0.73 ± 0.33	3.45 ± 0.033	15.23 ± 0.002	652 ± 2.25	96.13
F5	5.2 ± 0.33	0.95 ± 0.09	3.67 ± 0.043	15.10 ± 0.34	645 ± 3.30	96.71
F6	6.8 ± 0.42	0.67 ± 0.54	3.70 ± 0.022	15.28 ± 0.054	649 ± 4.25	99.03
F7	5.8 ± 0.21	0.54 ± 0.43	3.54 ± 0.011	15.19 ± 0.051	643 ± 4.32	98.13
F8	6.1 ± 0.22	0.62 ± 0.01	3.63 ± 0.012	15.22 ± 0.032	648 ± 4.24	99.01

Data shown as mean ± SD (n = 3).

**Table 6:** Floating behavior of metronidazole effervescent floating tablets.

Formulations	Tablet Density (g/cm^3^)	Floating Lag Time (sec)	Total Floating Time (h)
F1	0.984	NA *	NA *
F2	0.957	18	6
F3	0.975	12	>12
F4	0.987	4	>12
F5	0.993	NA *	NA *
F6	0.979	5	>12
F7	0.982	35	>12
F8	0.989	20	6

NA * represents no floating behavior of formulation; FLT of F2 and F8 vs. F4 and F6 (* *p* < 0.05), TFT of F2 and F8 vs. F3, F4, and F6 (* *p* < 0.05).

**Table 7:** Experimental pre-set conditions of dissolution apparatus.

Apparatus	USP Type Ⅰ (Basket)
Agitation speed	100 rpm
Medium	0.1 N HCl, pH 1.2
Volume	900 mL
Temperature	37 ± 1 °C
Time	0.5, 1, 2, 3, 4, 6, 8 and 12 h
Wavelength	277 nm

**Table 8:** Release kinetics of metronidazole floating effervescent tablets at 100 rpm.

Power Law				
Formulation Codes	K ± SD	R^2^	n	Release Mechanism
F2	0.001 ± 0.0088	0.9945	0.333	Does not follow power law kinetics
F3	0.001 ± 0.00142	0.9956	0.432	Quasi Fickian diffusion
F4	0.001 ± 0.00273	0.9978	0.52	Anomalous non-Fickian diffusion
F6	0.003 ± 0.007	0.9988	0.51	Anomalous non-Fickian diffusion
F7	0.001 ± 0.0078	0.9939	0.52	Anomalous non-Fickian diffusion
F8	0.001 ± 0.0014	0.9978	0.54	Anomalous non-Fickian diffusion

**Table 9:** Release kinetics of metronidazole floating effervescent tablets at 50 rpm.

Power Law				
Formulation Codes	K ± SD	R^2^	n	Release Mechanism
F2	0.001 ± 0.0013	0.9896	0.342	Does not follow power law kinetics (Fickian)
F3	0.001 ± 0.0016	0.9925	0.406	Quasi Fickian diffusion
F4	0.001 ± 0.0029	0.9965	0.50	Anomalous non-Fickian diffusion
F6	0.003 ± 0.007	0.9981	0.51	Anomalous non-Fickian diffusion
F7	0.001 ± 0.0008	0.9945	0.54	Anomalous non-Fickian diffusion
F8	0.001 ± 0.0017	0.9967	0.57	Anomalous non-Fickian diffusion

**Table 10:** Release kinetics of metronidazole floating effervescent tablets in 0.5% NaCl.

Power Law				
Formulation Codes	K ± SD	R^2^	n	Release Mechanism
F2	0.001 ± 0.067	0.9268	0.318	Does not follow power law kinetics
F3	0.001 ± 0.0008	0.9438	0.42	Quasi Fickian diffusion
F4	0.001 ± 0.0012	0.9937	0.42	Quasi Fickian diffusion
F6	0.001 ± 0.00146	0.9437	0.41	Quasi Fickian diffusion
F7	0.001 ± 0.0007	0.881	0.321	Does not follow power law kinetics
F8	0.001 ± 0.0006	0.9822	0.40	Quasi Fickian diffusion

**Table 11:** Release kinetics of metronidazole floating effervescent tablets in 0.9% NaCl.

Power Law				
Formulation Codes	K ± SD	R^2^	n	Release Mechanism
F2	0.001 ± 0.00028	0.9306	0.300	Does not follow power law kinetics
F3	0.0001 ± 0.00017	0.9591	0.44	Quasi Fickian diffusion
F4	0.001 ± 0.0003	0.9941	0.43	Quasi Fickian diffusion
F6	0.001 ± 0.0003	0.9499	0.44	Quasi Fickian diffusion
F7	0.0001 ± 0.00022	0.8792	0.278	Does not follow power law kinetics
F8	0.0001 ± 0.00014	0.9981	0.45	Quasi Fickian diffusion

## Data Availability

Not applicable.
